# Leukotriene D_4_ Upregulates Oxidized Low-Density Lipoprotein Receptor 1 and CD36 to Enhance Oxidized LDL Uptake and Phagocytosis in Macrophages Through Cysteinyl Leukotriene Receptor 1

**DOI:** 10.3389/fphys.2021.756450

**Published:** 2021-11-18

**Authors:** Sabita Pokhrel, Ravindra Gudneppanavar, Lakshminarayan Reddy Teegala, Ernest Duah, Charles K. Thodeti, Sailaja Paruchuri

**Affiliations:** ^1^Department of Chemistry, University of Akron, Akron, OH, United States; ^2^Department of Physiology and Pharmacology, University of Toledo College of Medicine and Life Sciences, Toledo, OH, United States; ^3^Department of Integrative Medical Sciences, Northeast Ohio Medical University, Rootstown, OH, United States

**Keywords:** LTD_4_, CysLT_1_R, phagocytosis, oxLDL, CD36, OLR1, MCP-1, atherosclerosis

## Abstract

Endothelial permeability, leukocyte attachment, and unregulated oxidized LDL (oxLDL) uptake by macrophages leading to the formation of foam cells are all vital in the initiation and progression of atherosclerosis. During inflammation, several inflammatory mediators regulate this process through the expression of distinct oxLDL binding cell surface receptors on macrophages. We have previously shown that Leukotriene D_4_ (LTD_4_) promotes endothelial dysfunction, increasing endothelial permeability and enhancing TNFα-mediated attachment of monocytes to endothelium, which hints at its possible role in atherosclerosis. Here we analyzed the effect of LTD_4_ on macrophage function. Macrophages mainly express CysLT_1_R and flux calcium in response to LTD_4_. Further, LTD_4_ potentiates phagocytosis in macrophages as revealed by the uptake of zymosan particles. Notably, LTD_4_ augmented macrophage phagocytosis and oxLDL uptake which is sensitive to MK-571 [Montelukast (MK)], a CysLT_1_R-specific antagonist. Mechanistically, LTD_4_ upregulated two receptors central to foam cell formation, oxidized low-density lipoprotein receptor-1 (OLR1/LOX-1), and CD36 in a time and dose-dependent manner. Finally, LTD_4_ enhanced the secretion of chemokines MCP-1 and MIP1β. Our results suggest that LTD_4_ contributes to atherosclerosis either through driving foam cell formation or recruitment of immune cells or both. CysLT_1_R antagonists are safely being used in the treatment of asthma, and the findings from the current study suggest that these can be re-purposed for the treatment of atherosclerosis.

## Introduction

Macrophages are innate immune cells present ubiquitously in the body, and they are involved in the phagocytosis of foreign materials and pathogens (Han et al., 2016). The role of macrophages is not only limited to engulfing foreign allergens, but also extends to ingesting self-antigens like extracellular debris and modified lipids (Patten and Shetty, 2018). Macrophages encounter diverse antigens, and they need distinct receptors to recognize them and initiate phagocytosis (Kelley et al., 2014). Phagocytosis is mediated through scavenger receptors classified into different groups ranging from A–J (Aderem and Underhill, 1999). Scavenger receptors not only function in scavenging self-antigens expressing damage associated molecular patterns (DAMPS) (Patten and Shetty, 2018), they also facilitate phagocytosis of particles like oxLDL that are the products of oxidative stress (Woo et al., 2016). Receptors like class B scavenger receptor CD36, Scavenger Receptor A (SR-A), CD204, and lectin like oxidized low density lipoprotein receptor (OLR1) in macrophages facilitate the internalization and degradation of modified lipids (Woo et al., 2016; Arslan et al., 2017), which initiates the buildup of foam cells, an event that is crucial in the initiation and progression of atherosclerosis. Atherosclerosis is an inflammatory disease involving endothelial dysfunction and the dysregulated uptake of lipid molecules into the blood vessels (Hansson and Hermansson, 2011). The accumulation of foam cells results in the formation of atherosclerotic plaques that further release their lipid contents into the vasculature. Plaque instability and its ultimate rupture results in the formation of a pro-thrombotic necrotic core during atherogenesis (Tabas and Bornfeldt, 2016). Macrophages are the key effector cells, and they have been extensively studied with respect to the disease (Moore et al., 2013). Attenuation of atherosclerotic complications in mice was observed when macrophages were egressed from the lesion microenvironment or when their phenotype was switched to resolution (M2) subset from their inflammatory (M1) counterparts (Feig et al., 2011a, b). Therefore, it is important to understand how soluble factors secreted during inflammation affect macrophage behavior, impacting atherosclerosis progression. From the time a link between inflammation and atherosclerosis was proposed, a range of inflammatory mediators were investigated for their possible role in this disorder (Nguyen et al., 2019). Increased expression of 5-lipoxygenase (5-LO) products, including leukotrienes and their receptors, were reported in atherosclerotic lesions, identifying these molecules as potential therapeutic targets for the disease (Back, 2009). Cysteinyl leukotrienes (cys-LTs) comprising of LTC_4_, LTD_4_, and LTE_4_ are derivatives of arachidonic acid generated by mast cells, macrophages, eosinophils, and basophils (Kanaoka and Boyce, 2004). Cys-LTs are the most potent bronchoconstrictors (Davidson et al., 1987; Drazen and Austen, 1987), and they are involved in the pathophysiology of various inflammatory diseases like asthma, rheumatoid arthritis, and cardiovascular diseases (Chung, 1995; Busse, 1996; Liu and Yokomizo, 2015). Cys-LTs mediate their biologic functions mainly through two known G protein-coupled receptors (GPCRs), CysLT_1_R, and CysLT_2_R (Lynch et al., 1999; Heise et al., 2000). Apart from these two main receptors, GPR17 is activated by LTD_4_ and acts as a negative regulator for CysLT_1_R (Maekawa et al., 2009). Further, LTE_4_, the most abundant and stable of the cys-LTs, is a weak, partial agonist for the CysLT_1_R and CysLT_2_R (Evans, 2002). In contrast to LTD_4_, LTE_4_ relays signals through both peroxisome proliferator activating receptor (PPAR)-γ, a ligand-activated transcription factor (Paruchuri et al., 2008), and P2Y_12_ receptor (P2Y_1__2_R), a GPCR that recognizes adenosine diphosphate (ADP) (Paruchuri et al., 2009). Recently, GPR99 was identified as another CysLTR with a preference for LTE_4_ (Kanaoka et al., 2013). Pro-inflammatory mediators generated during inflammation activate endothelial cells (EC) and leukocyte extravasation. Injection of each of the three cys-LTs has been shown to enhance dermal vascular permeability in mice and humans (Soter et al., 1983; Maekawa et al., 2008; Kondeti et al., 2013). We recently demonstrated that EC CysLT_2_R mediates calcium influx, EC contraction *in vitro*, permeability of blood vessels, as well as angiogenesis *in vivo* (Duah et al., 2013, 2019). In addition, we also demonstrated that cys-LTs enhance TNFα-mediated up-regulation of vascular cell adhesion molecule (VCAM-1) and also enhance the attachment of monocytes to the endothelium (Duah et al., 2013). Since CysLTR signaling causes endothelial dysfunction, leading to enhanced vessel contraction and permeability facilitating monocyte attachment to endothelium, we explored their role in regulating macrophage function in the current study. While there have been many studies on macrophages, foam cell formation, and atherosclerosis, the involvement of cys-LTs or associated molecular mechanisms in macrophage function impacting atherosclerosis progression is elusive. Therefore in this study, we analyzed the role of cys-LTs in the uptake of oxidized LDL by macrophages, an initial step in the formation of foam cells, and the mechanism involved.

## Materials and Methods

### Animals

Bone marrow-derived macrophages (BMDM) were cultured from wild type C57BL/6 (WT) mice (6–8-weeks old), purchased from the Jackson Laboratory and maintained at the University of Akron Research vivarium (UARV). Animals were euthanized in accordance with standard guidelines, as approved by the Animal Care and Use Committee of UA.

### Materials

Murine recombinant colony stimulating factor (m-CSF) was purchased from Peprotech (Cranbury, NJ). LTD_4_ and MK571 (MK) were from Cayman Chemicals (Ann Arbor, MI). Fura-2 AM was purchased from Molecular Probes (Eugene, OR). Texas-red conjugated zymosan bioparticles and DiI conjugated oxLDL were purchased from fisher scientific (Waltham, MA).

### Cell Culture

Raw 264.7 (raw) cells were cultured in Dulbecco’s Modified Eagle’s high glucose medium (DMEM; Corning, NY) supplemented with 10% FBS and 1% pen-strep. THP-1 monocytes were cultured in RPMI-1640 medium supplemented with 10% FBS and 1% pen-strep. These cells were differentiated into macrophages for 48 h in the presence of 50 ng/ml phorbol-12-myristate-13-acetate (PMA). For BMDM, bones (tibia and femur) were collected from 6 to 8 weeks old WT, *Cysltr1^–/–^*, and *Cysltr2^–/–^* mice on C57BL/6 background, and bone marrow cells (BMCs) were isolated by flushing bones. Cells were suspended in R10 media (RPMI-1640 supplemented with 10% FBS, 5% non-essential amino acids, 1% pen-strep, and 50 μM β-mercaptoethanol) and maintained at 37°C. BMCs were differentiated into BMDMs using 10 ng/ml macrophage colony stimulating factor (M-CSF). On third day, the culture plate was replenished with fresh R10 medium containing 10 ng/ml M-CSF, and incubated for 3 more days. We confirmed the purity of the culture by F4/80 staining.

### Immunofluorescence

Raw macrophages were fixed with 4% paraformaldehyde solution, and permeabilised with 0.25% Triton X-100 for 15 min. Cells were washed twice with PBS, blocked with 10% FBS containing medium for 30 min and were stained with CysLT_1_R antibody for 1 h. Thereafter, the cells were washed twice in PBS and incubated with Alexa Fluor 488 goat anti-rabbit secondary antibody for 45 min. Images were obtained using EVOS fluorescence microscope.

### Ca^2+^ Flux Assay

Raw cells, THP-1-derived macrophages, and BMDMs were loaded with Fura-2 AM for 30 min and washed in calcium buffer. Cells were stimulated with LTD_4_ (0.5 μM) in the presence or absence of CysLT_1_R antagonist MK (1 μM, 30 min pre-incubation). Changes in the intracellular calcium levels were measured using the ratio of excitation wavelengths (340/380 nm) in a fluorescence spectrophotometer (Hitachi F-4500).

The relative ratios of fluorescence emitted at 510 nm were recorded and displayed as a reflection of intracellular calcium concentration (Paruchuri et al., 2008).

### Zymosan Phagocytosis Assay

Macrophages were cultured as mentioned earlier, and 50,000 cells were plated in each well of an 8-well chamber slide in 200 μl DMEM high glucose, supplemented with 10% FBS, and stimulated with LTD_4_ (0.5 μM) for 24 h. Texas-red conjugated zymosan bioparticles were reconstituted to obtain uniform suspension according to the manufacturer’s protocol, and 500,000 zymosan bioparticles (1:10) were added to each well and incubated for 1 h. Excess zymosan particles were removed and washed with PBS, and imaged using a fluorescence microscope. The images were quantified by ImageJ and the percentage phagocytosis was calculated based on the percentage of number of cells with zymosan particles compared to total number of cells (DAPI staining).

### Oxidized LDL Uptake Assay

Macrophages were stimulated with 0.5 μM LTD_4_ for 24 h in the presence or absence of CysLT_1_R antagonist MK (1 μM) pre-incubated for 30 min. After 24 h, macrophages were incubated with oxLDL (10 μg/ml) for 1 h at 37°C in a humidified incubator with 5% CO_2_ environment and stained with oil red O (only stains the lipid particles). Excess stain was washed with PBS, and the slides were observed under the microscope. Quantification of phagocytosis was done using ImageJ (NIH) as described above.

### Real-Time Quantitative PCR

The expressions of mOLR1, mCD36, and mMCP-1 were determined with qPCR performed on Light cycler 480 (Roche) (Kondeti et al., 2016). Total RNA was isolated from Raw cells, THP-1-derived macrophages, and BMDMs after respective treatments with an E.Z.N.A. Total RNA kit 1 (Omega Bio-Tek, Norcross, Georgia). DNAse contamination was removed using a DNA-free DNA Removal Kit (Invitrogen, Waltham, MA) based on the manufacturer’s instructions. cDNA was synthesized using a cDNA synthesis kit (Roche, Indianapolis, IN). qPCR was performed using the primers mentioned below. The levels of respective genes relative to the GAPDH were analyzed, and the ΔΔCT values were calculated and expressed as relative expression or fold change compared to control (no template). The quality of the RNA, primers, and qPCR reaction was validated using proper controls, like no RT control or no template control. Real time PCR for each sample was performed in at least triplicates and then repeated in three different experiments.

#### Primers

***mOLR1***
F: 5′-ACAATACCAAGCGAACCTTACT-3′; R: 5′-TGGGT GAGGGTGTCTATCTT-3′
***mCD36***
F: 5′-CCAGTCGGAGACATGCTTATT-3′; R: 5′-GTACAC AGTGGTGCCTGTT-3′
***mMCP-1***
F: 5′-AGTAGGCTGGAGAGCTACAA-3; R: 5′-GTATGT CTGGACCCATTCCTTC-3′
***mGAPDH***
F: 5′-CTCCCACTCTTCCACCTTCG-3′; R: 5′-CCACCA CCCTGTTGCTGTAG-3′

### ELISA

The concentrations of MCP-1 and MIP1β secreted into the medium by macrophages after respective treatments were analyzed by MCP-1 ELISA kit (Invitrogen, Waltham, MA) and MIP1β ELISA kit (R & D Systems, Minneapolis, MN), respectively, according to the manufacturer’s protocol (Kondeti et al., 2016).

### Statistical Analysis

Data are expressed as means ± SEM from at least three experiments except where otherwise indicated. Data were converted to a percentage of control for each experiment where indicated. Significance was determined using one-way ANOVA, and comparisons between the groups were determined by Tukey’s multiple comparisons test (GraphPad Prism 7.01; GraphPad Software, La Jolla, CA, United States). *P < 0.05, **P < 0.01, ***P < 0.001.

## Results

### Leukotriene D_4_ Mediated Calcium Flux in Macrophages

**FIGURE 1:**
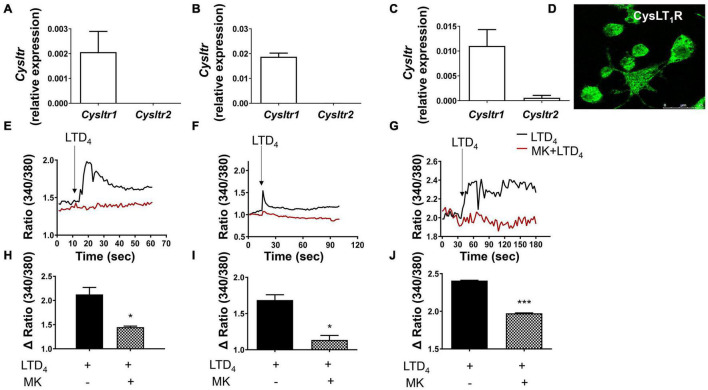
Cys-LTs induce calcium flux in macrophages through CysLT_1_R. The expression of CysLT_1_R and CysLT_2_R transcript was analyzed in **(A)** raw macrophages, **(B)** THP-1-derived macrophages, and **(C)** BMDMs by qPCR. **(D)** Immune-staining of raw macrophages for CysLT_1_R expression. Macrophages were loaded with Fura-2-AM, stimulated with LTD_4_ (0.5 μM), and then calcium flux was measured in **(E)** raw macrophages, **(F)** THP-1-derived macrophages, and **(G)** BMDM in the presence or absence of CysLT_1_R antagonist MK. Panels **(H–J)** represent quantification of data from panels **(E–G)**, respectively. The results shown are mean ± SEM from three independent experiments (Student’s *t*-test, *p ≤ 0.05 and ***p ≤ 0.001).

To understand the role of CysLTR signaling in regulating macrophage function, first we studied the expression of CysLT_1_R and CysLT_2_R in three different macrophage cell types- raw macrophages, THP-1-derived macrophages, and BMDMs by qPCR. Our results revealed that all macrophages mainly express CysLT_1_R compared to CysLT_2_R (Figures 1A–C). We observed a modest expression of CysLT_2_R in BMDMs. None of the macrophages revealed expression of GPR99 transcript (not shown). Immune-staining of raw macrophages revealed significant CysLT_1_R expression at protein level (Figure 1D). Further, in Fura-2 loaded macrophages, LTD_4_ induced robust calcium flux, which is completely blocked by pretreatment of the cells with MK (Figures 1E–J), which competitively antagonizes CysLT_1_R, but not CysLT_2_R (Paruchuri et al., 2008; Duah et al., 2019). Thus, macrophages flux calcium mainly *via* CysLT_1_R.

### Phagocytosis in Response to Leukotriene D_4_ in Macrophages

**FIGURE 2:**
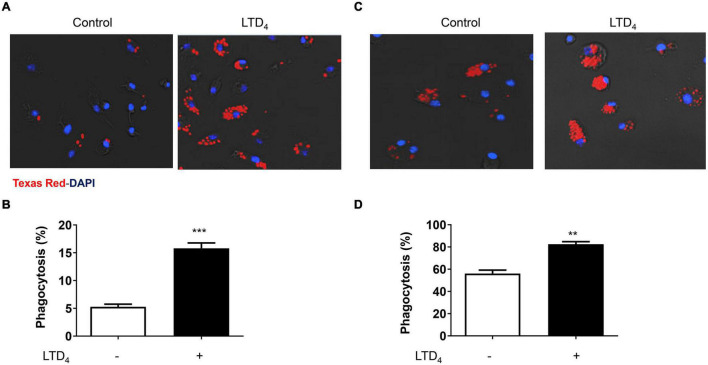
Cys-LTs enhance phagocytosis in macrophages. Fluorescence micrographs showing zymosan particle phagocytosis in **(A)** raw macrophages and **(C)** BMDMs. Macrophages were treated with 0.5 μM LTD_4_ for 24 h and incubated with zymosan particles (1:10) for 1 h. Images were quantified using ImageJ. Panels **(B,D)** represent the quantification of raw macrophages and BMDMs, respectively. The results shown are mean ± SEM from three experiments performed (Student’s *t*-test, **p ≤ 0.01 ***p ≤ 0.001).

To explore the phagocytic ability of macrophages in response to LTD_4_, we treated raw macrophages and BMDMs with 0.5 μM LTD_4_ for 24 h, and then performed phagocytosis assay using Texas red conjugated zymosan particles. LTD_4_ increased the phagocytosis of zymosan particles in raw macrophages (Figures 2A,B). Although BMDM exhibited higher basal phagocytosis compared to raw macrophages, LTD_4_ significantly potentiated phagocytosis in these macrophages (Figures 2C,D).

### Effect of Leukotriene D_4_ on Oxidized LDL Uptake in Macrophages

**FIGURE 3:**
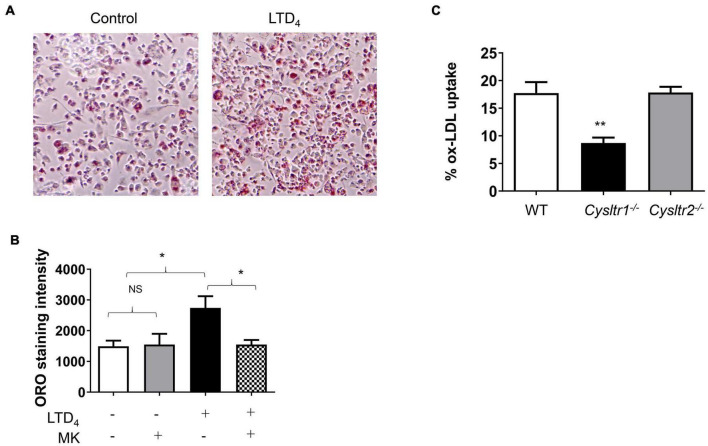
Cys-LTs induce oxLDL uptake in macrophages. Representative images showing oxLDL uptake in macrophages. Cells were treated with 0.5 μM LTD_4_ for 24 h in the presence or absence of CysLT_1_R antagonist MK (1 μM) and then incubated with oxLDL for 1 h. Thereafter, cells were stained with oil red-O (ORO), imaged and quantified using ImageJ. **(A)** Images of control and LTD_4_-treated cells depicting positive ORO staining, **(B)** quantification of ORO staining in macrophages pre-treated with or without MK and treated with or without LTD_4_. **(C)** Bone marrow cells were isolated from WT, *Cysltr1^– /–^*, and *Cysltr2^– /–^* mice and then cultured for 6 days with M-CSF to differentiate them into macrophages. BMDMs were incubated with fluorescently labeled oxLDL for 1 h and the oxLDL uptake was measured by analyzing the fluorescence incorporated into the cells using BioTek microplate reader. Results shown are mean ± SEM from three separate experiments (one-way ANOVA followed by *post hoc* Tukey multiple comparison test, *p ≤ 0.05, **p ≤ 0.01, ns = not significant).

To determine whether LTD_4_ can modulate the uptake of oxLDL, macrophages were subjected to LTD_4_ for 24 h followed by incubation with oxLDL for another hour. The uptake of oxLDL was determined by staining with oil red O. We observed enhanced uptake of oxLDL when macrophages were treated with LTD_4_, as visualized by oil red O staining (Figures 3A,B). Notably, CysLT_1_R antagonist MK abrogated this response, suggesting that LTD_4_ potentiates oxLDL uptake *via* CysLT_1_R. In agreement, BMDM lacking CysLT_1_R exhibited an attenuated oxLDL uptake compared to WT and CysLT_2_R-deficient BMDMs (Figure 3C).

### Leukotriene D_4_-Induced Changes in Oxidized LDL Receptors

**FIGURE 4:**
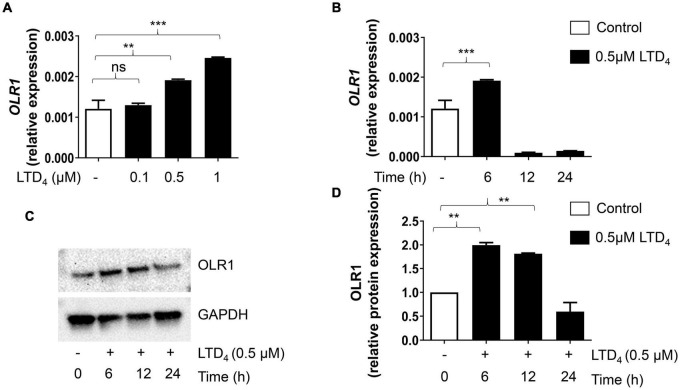
Cys-LTs induce upregulation of OLR1 transcript and protein. Raw macrophages were treated with increasing concentrations of LTD_4_ for 6, 12, and 24 h, and the expressions of *OLR1* transcript **(A,B)** was analyzed using qPCR and protein expression was analyzed by Western blotting **(C,D)**. The results shown are mean ± SEM from three separate experiments (one-way ANOVA followed by *post hoc* Tukey multiple comparison test, **p ≤ 0.01, ***p ≤ 0.001, ns = not significant).

**FIGURE 5:**
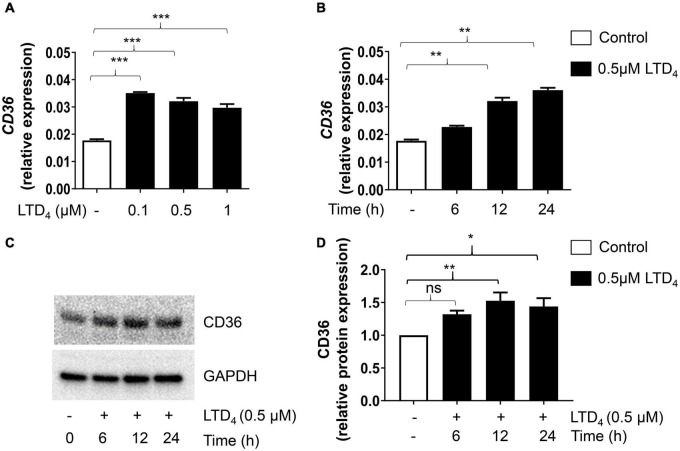
Cys-LTs induce upregulation of CD36 transcript and protein. Raw macrophages were treated with increasing concentrations of LTD_4_ for 6, 12, and 24 h, and the expressions of CD36 transcript **(A,B)** was analyzed using qPCR and protein expression was analyzed by Western blotting **(C,D)**. The results shown are mean ± SEM from three separate experiments (one-way ANOVA followed by *post hoc* Tukey multiple comparison test, *p ≤ 0.05, **p ≤ 0.01, ***p ≤ 0.001, ns = not significant).

Macrophages are known for their receptor-mediated phagocytosis to ingest extracellular particles (Guest et al., 2007). Because LTD_4_ enhances phagocytosis and oxLDL uptake, we examined if LTD_4_ promotes the expression of scavenger receptors. We treated macrophages with LTD_4_ and analyzed the mRNA expression of receptors known to be involved in phagocytosis by qPCR. *OLR1* transcript was upregulated with LTD_4_ in a dose-dependent manner (Figure 4A). Similarly, LTD_4_ caused up-regulation of CD36 transcript (Figure 5A), starting from 0.1 μM and sustained with increasing doses. Temporally, *OLR1* mRNA upregulation by LTD_4_ was relatively early, peaking at 6 h and declined later (Figure 4B). In contrast, CD36 transcript was enhanced starting 6 h and sustained till 24 h (Figure 5B). Reflecting our transcript data, we observed increase in OLR1 protein at 6 and 12 h of LTD_4_ treatment and declined by 24 h (Figures 4C,D). Similarly, CD36 protein expression is augmented by LTD_4_ treatment starting at 6 h with a significant increase at 12 and 24 h (Figures 5C,D).

### Induction of Monocyte Chemoattractant Protein-1 by Leukotriene D_4_

**FIGURE 6:**
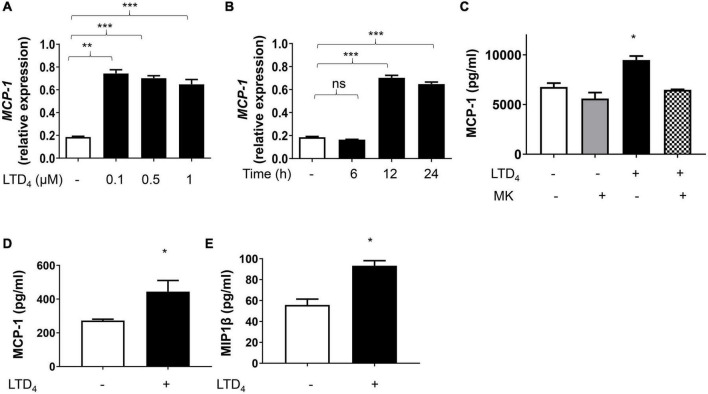
CysLTs enhance MCP-1 production in macrophages. Raw macrophages were treated with increasing concentrations of LTD_4_ **(A)**, for 6, 12, and 24 h **(B)**, and *MCP-1* transcript was analyzed using qPCR. In panel **(C)**, raw macrophages were pre-incubated for 30 min in the presence or absence of MK571, treated with 0.5 μM LTD_4_ for 6 h and supernatants were collected and analyzed for MCP-1. BMDMs **(D,E)** were treated with 0.5 μM LTD_4_ for 6 h, supernatants were collected and **(D)** MCP-1 protein and **(E)** MIP1β protein in the supernatants were analyzed by ELISA according to the manufacturer’s instructions. The results shown are mean ± SEM from three experiments (one-way ANOVA followed by *post hoc* Tukey multiple comparison test, *p ≤ 0.05, **p ≤ 0.01 ***p ≤ 0.001, ns = not significant).

MCP-1 (CCL-2) has been associated with atherosclerosis *via* increasing foam cell load in the intima of the blood vessels (Lin et al., 2014). We asked whether LTD_4_ induces MCP-1 expression by macrophages. Real-time PCR analysis showed that LTD_4_ stimulation of raw macrophages induced the expression of MCP-1 transcripts at all doses tested (Figure 6A). Further. LTD_4_-potentiated MCP-1 transcript peaked at 12 h and sustained till 24 h (Figure 6B). Consistent with mRNA data, LTD_4_ induced MCP-1 expression at the protein level as determined by ELISA, sensitive to MK571 (Figure 6C). Notably, we found similar potentiation of MCP-1 and MIP1β in BMDMs (Figures 6D,E).

## Discussion

5-Lipoxygenase metabolites have been implicated to play an important role in phagocytosis of macrophages (Serezani et al., 2011), and they are associated with inflammatory diseases like atherosclerosis (Back and Hansson, 2006). The 5-LO pathway has been demonstrated to be abundantly expressed in the arterial walls of patients suffering from various lesion stages of atherosclerosis of the aorta, with an increased number of 5-LO expressing cells (macrophages, dendritic cells, foam cells, mast cells, and neutrophilic granulocytes) in advanced lesions (Spanbroek et al., 2003). Notably, mice deficient in 5-LO were reported to exhibit reduced lesions in *LDLR^–/–^* background, suggesting that leukotrienes may play a dominant role in atherogenesis (Mehrabian et al., 2002). LTB_4_, also a 5-LO metabolite, was shown to play vital roles during atherogenesis *via* its receptors, BLT-1 and BLT-2 (Subbarao et al., 2004). Although the involvement of the 5-LO pathway in mediating atherosclerosis is convincing, the role of CysLTR and associated signaling in modulating macrophage function and atherosclerosis still remains elusive. Macrophages are not only equipped with all the essential enzymes to synthesize cys-LTs in response to various agonists, but also possess the relevant receptors to facilitate autocrine signaling. Therefore, it is vital to understand how cys-LTs modulate macrophage function. Previous studies from our lab suggest that CysLTR signaling causes endothelial dysfunction and potentiates the attachment of monocytes to EC in response to TNFα (Duah et al., 2013). Based on these findings, we speculated that cys-LTs generated at the site of inflammation may also trigger macrophage dysfunction and contribute to atherosclerosis. To address this, we first confirmed the CysLTR expression in three different macrophage populations. We found that macrophages mainly express CysLT_1_R compared to CysLT_2_R, in agreement with the literature (Lotzer et al., 2003). Since CysLT_1_R couples to Gαq in many systems, generating calcium flux upon activation (Lynch et al., 1999), we measured intracellular calcium in macrophages in response to LTD_4_ and confirmed that macrophages mainly flux calcium in response to LTD_4_ *via* CysLT_1_R, employing CysLT_1_R antagonist MK. We next asked what effect this receptor has in modulating macrophage phagocytosis. Macrophages play a vital role in the phagocytosis of infectious agents, pathogens, and debris during inflammation, which is crucial for maintaining cellular homeostasis (Han et al., 2016). We observed that LTD_4_ significantly promoted phagocytosis of zymosan bioparticles in both raw macrophages and BMDMs, although BMDMs exhibited enhanced basal phagocytosis compared to raw macrophages. Endothelial dysfunction leading to lipid modification is perceived as a danger signal by the macrophages, and they function by engulfing these cholesterol-rich lipid molecules, leading to the formation of lipid-laden foam cells (Tabas and Bornfeldt, 2016). Our previous study demonstrated that cys-LTs cause endothelial cell (EC) dysfunction such as EC contraction, gap formation, and attachment of monocytes to the endothelium (Duah et al., 2013). Notably, LTB_4_ (Zhang et al., 2017) and cys-LTs (Yu et al., 2014) have been shown to be involved in the recruitment of immune cells to the site of inflammation, enhancing phagocytosis. Further, the enhanced expression of 5-LO and cys-LTs have been shown in atherosclerotic lesions, suggesting their potential role in plaque instability and atherosclerosis progression (Qiu et al., 2006). Based on these studies, we wondered about the role of the LTD_4_/CysLT_1_R axis on oxLDL uptake in macrophages. Our results demonstrate that LTD_4_ *via* CysLT_1_R enhanced the uptake of oxLDL in macrophages. BMDMs lacking CysLT_1_R exhibited an attenuated uptake compared to WT and CysLT_2_R null BMDMs, further suggesting an important role of cys-LTs in engulfing oxidized lipids. We further explored the mechanism and relevant cell surface oxLDL receptors activated by LTD_4_, which are responsible for lipid accumulation and foam cells in macrophages. OxLDL acts *via* binding to several receptors, including CD36, and OLR1, Peroxisome proliferator-activated receptor-gamma coactivator 1 α (PGC-1α), and SRA mediating lipid accumulation (Febbraio et al., 2000; Guest et al., 2007; Patten and Shetty, 2018). We observed the upregulation of *OLR1* and *CD36* in response to LTD_4_. Notably, we could not detect the upregulation of other scavenger receptors like *PGC1*α and *SRA1* by LTD_4_ (not shown), suggesting that LTD_4_ signaling is relayed mainly *via* CD36 and OLR1, contributing to enhanced uptake of lipid molecules. OLR1 is a membrane glycoprotein that can selectively bind and internalize oxLDL (Ogura et al., 2009). Several inflammatory and atherosclerosis-related stimuli have been shown to induce OLR1 expression, including lipopolysaccharide (LPS), TNFα, interleukin-1 (IL-1), interferon gamma (IFNγ), oxLDL, and angiotensin II (Xu et al., 2013). CD36 belongs to the class B scavenger receptor family, and it is expressed on various cell types, including macrophages, platelets, and microvascular EC (Park, 2014). CD36-null mice were shown to exhibit increased cholesterol, triacylglycerol, and fatty acids in the plasma level, suggesting a major role of CD36 in fatty acid uptake and lipid metabolism *in vivo* (Febbraio et al., 1999).

Apart from the above mentioned receptors, chemokine CCL2/MCP-1 is a critical mediator of atherosclerosis, and the absence of MCP-1 has been shown to reduce atherosclerosis in low-density lipoprotein receptor-deficient mice (Gu et al., 1998). In support, MCP-1 expression was observed in human and rabbit atherosclerotic plaques (Yla-Herttuala et al., 1991), and a reduction in arterial lipid deposition was observed in CCL2 deficient mice (Boring et al., 1998). MCP-1 null mice were shown to have severe defects in monocyte recruitment to inflammatory sites (Gu et al., 1998), suggesting that MCP-1 plays an essential role in monocyte/macrophage populations. Interestingly, LTD_4_ was shown to up-regulate MCP-1 in human monocytes and macrophages (Ichiyama et al., 2005). This prompted us to analyze if LTD_4_ signaling to lipid uptake required MCP-1. We observed an enhanced *MCP-1* expression in response to LTD_4_, both at the transcript and protein level.

## Conclusion

**FIGURE 7:**
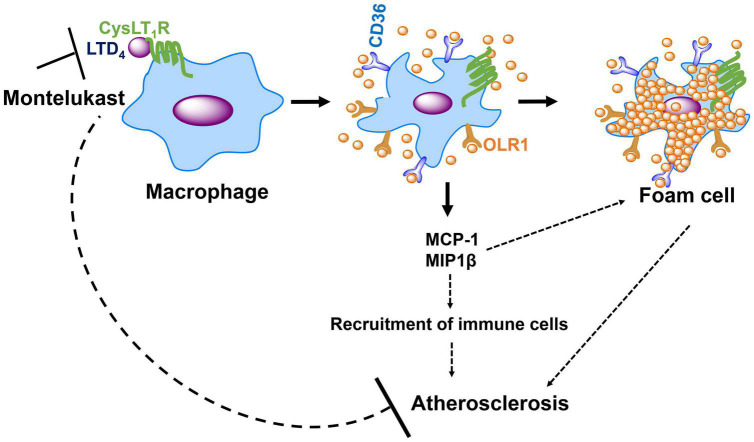
Schematic, suggests the role for CysLT_1_R in macrophage activation and atherosclerosis. LTD_4_ stimulation induces the upregulation of oxLDL receptors, OLR1, and CD36 *via* CysLT_1_R, which in turn facilitates oxLDL uptake in macrophages, resulting in foam cell formation. LTD_4_ also induces the secretion of MCP-1 and MIP1β in macrophages, which further recruits immune cells, amplifying inflammation. These events can lead to atherosclerosis, and our study suggests that CysLT_1_R antagonist, MK (Montelukast) can be used as a novel therapeutic target for the treatment of atherosclerosis.

In conclusion, our study demonstrated a role for cys-LT/CysLT_1_R in upregulating CD36 and OLR1 receptors and MCP-1, and subsequent uptake of oxidized lipid molecules (Figure 7). All these events are crucial for foam cell formation during atherosclerosis. CysLT_1_R antagonists are FDA-approved and have been widely used in the therapy of asthma for the past few decades, with minimal side effects. Our study further suggests that these drugs may be repurposed for the treatment of atherosclerosis.

## Data Availability Statement

The raw data supporting the conclusions of this article will be made available by the authors, without undue reservation.

## Ethics Statement

The animal study was reviewed and approved by the Animal Care and Use Committee of University of Akron.

## Author Contributions

SPo, RG, LT, and ED performed the experiments, analyzed the data, and edited the manuscript. CT designed the experiments and edited the manuscript. SPa designed the experiments, performed the research, analyzed and interpreted the data, and wrote the manuscript. All authors contributed to the article and approved the submitted version.

## Conflict of Interest

The authors declare that the research was conducted in the absence of any commercial or financial relationships that could be construed as a potential conflict of interest.

## Publisher’s Note

All claims expressed in this article are solely those of the authors and do not necessarily represent those of their affiliated organizations, or those of the publisher, the editors and the reviewers. Any product that may be evaluated in this article, or claim that may be made by its manufacturer, is not guaranteed or endorsed by the publisher.

## Abbreviations

CysLT_1_R: Cysteinyl Leukotriene 1 Receptor
CysLT_2_R: Cysteinyl Leukotriene 2 Receptor
BMDM: Bone marrow-derived macrophages
LTD_4_: Leukotriene D_4_
OLR1: Oxidized low-density lipoprotein receptor 1
LDL: Low density lipoprotein
MCP-1: Monocyte chemoattractant protein-1
MIP1 β: Macrophage Inflammatory protein-1 β.
